# Surgery

**Published:** 1859-09

**Authors:** 


### SURGERY.

1. ON CALCULOUS DISEASES IN HUNGARY. By *Professor Balassa.*—This article is the substance of a reply made by Professor Balassa, of Pesth, to a circular asking for statistical information, issued by Professor Gross, of Philadelphia, U. S. Owing to the absence of rural hospitals in Hungary, Professor Balassa observes, almost all the cases of stone which occur in Hungary are brought to Pesth for treatment; and in his clinic at the hospital, he has treated, in the twelve years, 1843–55, 135 cases. The ages of these patients were as follows:—

| | |
|---|---|
| From 1 to 7 years | 21 |
| " 8 " 15 " | 32 |
| " 16 " 25 " | 47 |
| " 26 " 50 " | 27 |
| " 51 " 60 " | 6 |
| " 61 " 70 " | 2 |
| | 135 |

The employments and conditions of these patients were as follows:—Peasants, 82; artisans, 39; tradespeople, 7; *employees,* 2; landowners, 2; students, 2; teacher, 1.

Thus calculous affections are met with in the Pesth clinic with by far the greatest frequency in the young, inasmuch as out of a total of 135 cases, 100 of the patients had not reached their twenty-sixth year; and when it is added that in most of the cases the disease had long existed, its origin must be referred to a still earlier age. Moreover, during the twelve years, there were also forty-nine children treated for stone, in the Pesth Children's Hospital. So, too, the immense proportionate prevalence of the disease in the peasant (60·74 per cent.) and in the artisan (28·889 per cent.) classes is to be remarked. These classes, indeed, almost exclusively furnish the examples of the disease occurring among the young, as the author's private practice has taught him. Dr. Ivanchich, too, in his statistics of 100 cases of stone, comprises 33 natives of Hungary, but only 2 of these are as young as eighteen. The conclusion is, then, that stone prevails especially among the young of the poorer classes; and this leads us to consider some of the influences that are at work in its production. The nature of the diet and mode of life can alone explain these differences. It is the custom in Hungary to feed children when weaned, or even while suckling, upon the same articles of diet as are employed by adults, and while these in the wealthy classes may be nutritious and digestible, among the poor they consist of unwholesome matters, as fruits, vegetables, pork, and bacon. The latter, then, are fed with a diet that is difficult of digestion, and containing by far too large a proportion of the carbonaceous element; and the importance of this statement will be seen when the chemical constitution of the calculi has been considered.

For various reasons, the author has only been able to preserve 83 calculi in his cabinet, but all these have been carefully examined, and he furnishes the details of the results: 63 of the calculi were of homogeneous composition, and in 20 the nuclei and external portions were of different composition. The general result is, that while uric acid was the most frequent constituent, it was not the most frequent chief constituent, for while it was found more or less in 72 calculi, it constituted the chief portion of these only in 23. The oxalate of lime was the chief constituent; for it formed the chief mass of 20 calculi of homogeneous composition, and formed the nuclei in 12 out of the 20 stones of non-homogeneous composition. It is evident, then, that the formation of stone in Hungary is much due to the richness of the urine in oxalates, *i.e.* in the carbonaceous combinations furnished by the defective aliment employed. Of the 20 stones having nuclei of different composition to the surrounding parts, in 12 these consisted of oxalates and in 8 of urates. It is owing to the prevalence of these in the urine that the first impulse to the formation of calculi is given, while their enlargement much depends upon the presence of phosphates. These were present either as a chief or partial constituent in 45 calculi. These considerations lead to the conclusion that the most effectual means of preventing the formation or recurrence of calculi would be to act upon the oxalic or uric acid formations of the urine.

Of the 135 cases, 13, on account of disease of the urinary organs or the condition of the entire economy, were deemed unsuitable for operation. Some of the 122 operations were performed under very unfavorable circumstances, in consequence of the presence of chronic disease of the urinary organs.

Of the 122 cases operated upon, 92 were submitted to lithotomy, and 30 to lithotrity. Of the former, 11 patients, (11·95 per cent.,) and of the latter 5 (11·66 per cent.) died. Among the 11 fatal lithotomy cases, there were 5 individuals who suffered from severe chronic disease of the kidney, and one who died of typhus during an epidemic. There were, therefore, only 5 cases in which death took place in from the second to the fifth day, from inflammation of the bladder or peritoneum, consequent upon the operation. This reduces the strict mortality from the operation to 5·43 per cent. Among the 5 fatal lithotrity cases, in 1 death was due to phthisis, in 2 to old suppurative nephritis, and in 1 to recent nephritis, this last and one other death being alone directly referable to the operation, *i.e.* 6·66 per cent. Rectal fistula and urinary infiltration were never met with in any of the lithotomy cases. The difficulty in extracting the stone was considerable in many cases on account of its size, and in several of these inflammation of the bladder and peritoneum was set up, this proving fatal in 5 instances. Irritation and inflammation of the bladder frequently also followed lithotrity, leading to considerable delay in the repetition of the operation. With the exception of 7 cases, the stone was always removed entire. The largest calculus measured two inches five lines in diameter, in 5 calculi the diameter was above two inches, and in 24 between one and one and a half inch. The heaviest, removed from a boy ten years old, weighed one ounce and a half and ten grains, and the lightest seventeen grains. In 8 patients there were two stones, and in one three. In 2 children union by the first intention took place, and they left between the eighth and tenth day. The other patients were discharged between the twenty-first and sixtieth days. Relapse occurred in 2 instances after lithotrity, and in 1 after lithotomy.

Of the whole 135 cases only 1 occurred in a female, upon whom lithotrity was performed.

As to the mode of performing lithotomy, Professor Balassa makes an aperture in the bladder with a convex scalpel sufficiently large to admit the index finger of the left hand, and then enlarges it by means of a straight probe-pointed bistoury or Heister's knife. He lays great stress upon the wound being made sufficiently large, and in cases of voluminous calculi frequently makes a bilateral incision. After bleeding has been arrested by the injection of iced water, (performed while the fingers maintain the wound of the bladder open,) some small strips of oiled linen are carried along the index finger to the wound in the bladder, (especially when extraction has been difficult,) in order to prevent the urine penetrating the swollen edges of the track of the incision. They are removed after two or three days, and the author attributes the non-occurrence of infiltration principally to their employment. — *Wien Medizin. Wochenschr.*, 1858, Nos. 25 and 26.—*British and Foreign Medico-Chirurgical Review*, July, 1859.)

2. ON COLORING THE LIPS BY TATTOOING AFTER CHEILOPLASTY. By *Professor Schuh.*—Two years since Professor Schuh performed cheiloplasty in the Vienna Clinic, upon a girl in whom one-half of the nose, together with the vomer and the whole of both lips, were wanting. The flaps for the lower lip were supplied from the region of the lower jaw and the neck, and that for the nose from the forehead, while the skin of the arm was employed for the upper lip. The connection of the flap with the arm was divided on the tenth day, and all went on well, excepting that the new upper lip, at its lower edge, owing to the cicatricial process, was covered with corion. The red-lip color was wanting to give the mouth an agreeable appearance; and Professor Schuh determined to endeavor to imitate this by tattooing. He first of all tried cochineal as a coloring material, but this produced a too pale red, and he then had recourse to cinnabar, which gave rise to a surprisingly natural color.

The following is the procedure: the cinnabar is made into a thin paste with water, and the limits within which the pigment is to be applied are traced with a pen and ink, in imitation of the direction of the natural redness of the lips. For forcing the pigment into the organic substance, a bundle of sharp-pointed pins is employed, each pin being wound round with waxed silk from its head to within four lines of its point. Ten or twenty such pins are tied into a bundle with thread, dipped into the coloring substance, and repeatedly forced two or three lines deep into the lip. The margin marked by the ink is first to be colored, and then the other portion, dipping the points into the pigment again as this is wiped off. Only a slight bleeding ensues, and the pain is very little, in consequence of the diminished sensibility of transplanted parts. Any of the pigment remaining on the surface should be left there until next day, and if any part is found to be less red than the others this can be easily remedied.

How long this redness will remain unchanged must be determined by further experience. In Professor Schuh's case it had become nowise paler at the end of a year and a half; and he believes that the introduction of the process of tattooing into the field of plastic surgery is not to be despised.—(*Wien Medizin. Wochensch.*, 1858, No. 47.—*British and Foreign Medico-Chirurgical Review*, July, 1859.)

3. DIFFERENT MODES OF PERFORMING LITHOTOMY IN THE ENGLISH HOSPITALS. A large majority of English surgeons employ the ordinary lateral method of lithotomy on a curved staff. There has been, however, a considerable disposition to endeavor to improve it of late years. The median plan, so strongly recommended by Mr. Allarton, has been tried by not a few London surgeons, and among provincial ones has found a warm advocate in Mr. Teale, of Leeds. At the London Hospital it was first adopted by Mr. Ward, about two years ago, and since then has been employed by his colleagues, Mr. Critchett and Mr. Gowlland, each in a single instance. All the three patients were children, all recovered well, and in all it was considered that much less than the usual amount of bleeding took place. At Guy's Hospital, Mr. Cock has performed median lithotomy several times, and Mr. Erichsen has done the same at University College hospital, both surgeons being, we believe, well satisfied with its results. On all hands it is considered to be best adapted for children and for small stones. At St. Bartholomew's, Mr. Lloyd still continues to operate in all cases by his recto-urethral (median) method, which we described in detail when he first adopted it in 1853. He informs us that he has not yet lost a case after it, and considers it decidedly preferable to the lateral operation. His colleagues, however, without exception, we believe, always employ the latter. At the Metropolitan Free, Mr. Hutchinson always employs his rectangular catheter-staff, and considers that he obtains great advantage from it. The same instrument has been employed at King's College by Mr. Lee, but it is not, as far as we observe, in use at any other hospitals. In a recent instance in which the calculus was of large size, Mr. Hutchinson injected the bladder with oil, instead of water, in the hope of facilitating the dilatation of the parts.

With regard to the median operation as advised by Mr. Allarton, it is universally admitted to be adapted only for small calculi. Now Mr. Lloyd's experience during the last few years has quite proved that when the anterior commission of the sphincter ani is cut clean through from the perineal wound, there is no danger of the parts not healing. Might it not be well, therefore, to adopt this measure, whenever, after the usual median incisions, the stone has been reached and is found too large for removal? Mr. Lloyd's operation gives abundance of room.—(*Medical Times and Gazette*, July 9, 1859.)

4. ON GONORRHŒAL RHEUMATISM. By *M. Rollet*.—Notwithstanding what has been written on the subject, this affection is not universally admitted; and Professor Thiry, of Brussels, has recently formally denied its existence—there being, in his opinion, no rheumatism that can be legitimately called gonorrhœal, but only instances of arthritis coinciding with gonorrhœa, without any relation of cause and effect. M. Rollet, of Lyons, has, however, just published a memoir, in which he maintains the reality and peculiarity of this form of rheumatism. First, the frequency with which gonorrhœal subjects become affected with rheumatism is greater than it would be if it were a mere coincidence; and next, there is the repetition of the rheumatism so often met with in a given individual on the occurrence of a new, or the revival of an old gonorrhœa. While, too, it resembles ordinary rheumatism, it differs from it markedly not only in its cause, but in the fact of its rarely affecting the muscles, nerves, or viscera, and, above all, the heart. Another remarkable difference consists in its usually being mono-articular, or at least extending to but very few joints—the knee-joint being that which is much the most frequently attacked, and then the ankle, the other joints suffering much seldomer. Then, again, this form of rheumatism is often accompanied by an iritis, the seat of which is preferentially the anterior layer of the iris, and sometimes the serous membrane lining the posterior surface of the cornea, *i.e.* Desmours' membrane, either wholly or in part.

In the absence of pathological examination, it is difficult to say in what the lesions of gonorrhœal rheumatism differ from those of ordinary rheumatism; but there can be little doubt that they are not identical. At all events, it is certain that the inflammation becomes much more specially and more strongly fixed in the synovial membrane in this form of rheumatism than in the other, and hence the marked tendency to hydrarthritis which has struck all observers; and when it does not affect the joints, it exhibits a preference for the serous membranes, the sheaths of tendons, accidental brusæ, and Desmours' membrane. Clinically, gonorrœhal rheumatism is distinguished by the less acuteness of the inflammatory symptoms, whether general or local, and by a less tendency to suppuration; but, on the other hand, there is a greater tendency to anchylosis, especially in the small joints, and in predisposed subjects, to white-swelling. The disease, again, is excessively rare in women, if it is observed in them at all.

Gonorrhœal rheumatism seems to depend upon its special causes; for the predisposing and determining causes of common rheumatism have seemed to be without influence in the great majority of cases. The abundance of the discharge appears to be the most general condition upon which its production depends. Prior to the rheumatism, the discharge has usually been acute, and sometimes very acute; or having become chronic, has revived again. The duration of the discharge does not seem to exert the same influence. When the rheumatism has become developed, it exerts a repulsive effect upon the discharge, thus usually sensibly diminishing, although in a third of the cases it remains stationary. M. Rollet has seen it become more abundant only twice, and suppressed but once. The doctrine of metastasis has no foundation; and in treating the disease we must not seek to reproduce the discharge, as this doctrine would indicate; but, on the contrary, endeavor to arrest it as rapidly as possible, inasmuch as owing to the dependence of the rheumatism upon the urethritis the slightest exacerbation of the latter might be attended with aggravation of the synovitis. Therefore, copaiba and cubebs, together with injections or cauterization of the canal, should be resorted to. For the rheumatism itself, bloodletting is useful, local bleeding being, in the majority of cases, preferable. When the acute inflammation has been subdued, or if this never existed, and when articular effusion is the predominant feature, the flying blister is a most effectual means. Emetics and purgatives are valuable remedies, as are also vapor baths, especially toward the end of the malady.—(*Bulletin de Thérap.*, tome lv. p. 130.—*Medical Times and Gazette*, July 9, 1859.)

5. CANCER OF THE TONGUE; REMOVAL BY THE ECRASEUR.—[The écraseur has become almost obsolete in London hospital practice. We have ever looked upon its use as an unsurgical proceeding. Like every other novelty, it has had its trial, and it will soon be altogether laid aside, unless in some very exceptional surgical maladies, in which the risk of hemorrhage may again require its aid.]

On the twenty-first of June, a man of about forty-five years of age, with an epithelial cancer on the left side of the tongue, of four months' growth, had it removed in the Westminster Hospital, by Mr. Brooke, by means of this instrument. The cancerous mass was of the size of a walnut. The centre of its base was pierced with a large needle, and the chain of the écraseur drawn through it. The left half of the tumor was very rapidly divided by the instrument, and then the other half, thus taking away the entire disease in one piece, leaving a healthy surface behind. There was not much bleeding, although one or two small vessels required to be tied. The patient was fully under the influence of chloroform while undergoing the operation, and since its performance he may be said to have been going on very satisfactorily. Mr. Brooke stated that his reason for using the écraseur, in preference to the knife, was, to avoid the hemorrhage, which would not be so great by thus tearing the vessels asunder with this instrument.—(*Lancet*, July 2, 1859.)

6. POPLITEAL ANEURISM TREATED BY FLEXURE OF THE LIMB AND COMPRESSION; RUPTURE OF THE SAC INTO THE KNEE-JOINT; LIGATURE OF THE ARTERY; CURE.

(Under the care of *C. H. Moore, Esq.*)—The following case appears to us of very great interest in many points of view. It illustrates the great difficulty of successfully applying the treatment by compression when the patient is very muscular, and the tumor of large size and growing rapidly, and it is a forcible example of one of the most formidable dangers of certain forms of aneurism in the ham, viz., ulceration of the ligament separating the sac from the cavity of the knee-joint, and the effusion of the contents of the sac into that cavity. This accident is most liable to occur when the opening in the artery is situated on its anterior side, and that this was the case in this instance was rendered probable, in Mr. Moore's opinion, not merely by the subsequent rupture into the joint, but also by the thrilling character of the pulsation previously. We have ourselves observed similar cases, in which the wave of fluid in the vessel appeared to be communicated to the hand distinct and superficial to the more general heaving of the aneurismal tumor, and have noticed in such cases the increased liability of the joint to irritation and to synovial effusion. A slight increase of the same action which leads to this effusion will produce ulceration in the walls of the aneurism, already sufficiently disposed to it, if there is no great amount of clot, and thus the joint is laid open. It will be noticed that the treatment by flexion of the joint, which was recently brought before the Medico-Chirurgical Society by Mr. Ernest Hart, and which succeeded so perfectly in his case, and in one under the care of Mr. Shaw, which was read at the same meeting, was adopted here, but was not persevered in, as it evidently did no good, and may even, perhaps, by producing some direct pressure forward on the tumor, have tended to increase its liability to implicate the joint. We may conjecture, from the relations of the parts and from the shape which the artery would assume when, on the one hand, stretched over an aneurismal tumor seated behind or superficial to it, and on the other, relaxed behind one situated on its opposite side, that this treatment by simple position will be found adapted mainly, if not exclusively, to the former class of aneurisms, and probably will succeed only in those which are of moderate size and not growing very rapidly.

The perfect success of the ligature in this case, both in curing the aneurism and restoring the knee-joint to its functions, ought always to be kept in mind in the treatment of a similar grave complication. Even although so perfect a success might not occur in a second case, it would certainly be the best practice to give the patient the chance of the ligature of the artery before sacrificing the limb, and to watch for any indication of mischief set up by the clot in the knee-joint. The occurrence of abscess in the joint, as a result of such irritation, would probably limit the efforts of conservative surgery, and amputation would then be practiced as the last resource. Such was the view taken by Mr. Moore of the present very fortunate case—for the notes of which we are indebted to his case-book.

A fine, manly, highly-nourished navigator, aged forty-six, was admitted on January 18th, 1859, under Mr. Moore's care, with a very large aneurism in the right ham. It reached five inches and a half in vertical extent, and had its greatest lateral breadth at the level of the knee-joint; its highest part lay under the vastus internus, in the course of the artery; while below it extended a little way beneath the gastrocnemius. The aneurism expanded visibly, and with great force, in every direction, but more so laterally than backward; and its beat was accompanied by a delicate thrill against the hand laid on the popliteal space, and with a distinct systolic and feeble diastolic *bruit*. There was no swelling of the knee-joint, but the leg and foot were œdematous. The artery in the thigh beat with great force; those in the leg and foot very feebly. The current in the femoral being stopped, the aneurism became soft, and did not appear to contain much clot; it resumed its full size again in about two pulsations.

The history was, that about five or six weeks previously, without any particular injury or strain, his knee and whole leg swelled very greatly, and occasioned him much pain. He was directed to foment the limb; and much of the swelling subsided. Three weeks ago he felt the pulsation. The limb, at the time of admission, was useless; but he did not think that the knee was enlarging.

The knee was flexed, and fastened in that position with webbing passed in a figure of 8 around the foot and thigh; a pad and tight roller being inserted under the webbing, to moderate the flow of blood through the femoral artery. This left the aneurism beating slightly, but not visibly; and the *bruit* still audible in it, although less loud.

Next day the bandage having slipped, and being apparently of little service, was more firmly applied; and a rib-roller was added round the trunk, and passed in a figure of 8 around the foot. The result of all this was, that the aneurism beat comparatively little. The limb did not become materially gorged in the lower part.

January 20th. The aneurism being a very large one, and the webbing having slipped in the night, and rather chafed the foot, Mr. Moore readjusted the sling; but also applied over it a Skey's tourniquet, and instructed the man in the use of it.

On January twenty-first, it is noted that the aneurism had a decidedly less firm pulsation. He used the tourniquet steadily during the day, and the limb became gorged and œdematous below it. The force of the pulsation appeared, however, in the evening, to be increasing again; and the aneurism to be growing upward beneath the vastus internus. The webbing was readjusted, Skey's tourniquet was put above it, and the Italian tourniquet below, with orders to tighten them alternately.

Compression was continued during the next ten days, but the flexion of the limb was given up after a short time, and the foot raised upon a double matrass. Œdema of the limb, however, came on; and yet the powers of the pulsation did not diminish. It is stated that, notwithstanding every care on the part both of the patient and his medical attendants, the tourniquets did not act permanently well.

On January thirty-first, when he was seen, the shape of the knee was at once observed to be altered, the synovial membrane being full, and the patella raised from the condyles. It could be pressed down through some fluid. On loosening the tourniquet, pulsation was found in the joint, but in no other part of the limb except the aneurism; so that it was clear that the aneurism had burst into the joint. When the tourniquet was loosened, the joint became full and tense; and this tension was diminished again when the pulsation was cut off.

A consultation was now held; and it was agreed that the artery should be tied, in the hope that the opening between the sac and the joint might be obliterated, and the blood then absorbed from the joint. It was the more likely that this would occur, since the opening could have been only very recent, as the change in shape had not been noticed by the house-surgeon at his morning visit.

The artery was accordingly tied, at 3 P.M., by Mr. Moore. The wound was made large and very oblique. No difficulty was experienced in finding the artery, which was tied with a strong silk ligature. The knee-joint did not become flaccid after the operation, nor could its contents be squeezed out again.

In the evening, he was found very comfortable. The toes were cold, but not more so than those of the other limb; the calf and heel slightly warmer. A liberal diet was allowed, and the limb was kept for the next eight days encircled in flannel bandage. No sloughing followed the operation. The wound suppurated a little, and the ligature did not come away till February twenty-first. On the removal of the bandage, on February eighth, the knee was found of its natural form, and without blood. The aneurism was then decidedly smaller, circumscribed, and firm. The knee measured, round the top of the patella, seventeen inches and one-eighth, being five-eighths of an inch less than on his admission. On February twenty-first, when the ligature came away, it is noted that the convex rounded part of the tumor was soft, and fluctuated with a readiness almost like that of pus. Under the use, however, of astringent ointments, sal ammoniac, and tannin, the tumor lost this almost fluid softness, and much diminished in size. He was discharged in April.

On May twelfth he returned, able to bend his knee to a right angle, and having walked to the hospital from Hampstead. The ham merely seemed full, and it was necessary to flex the knee before any tumor could be felt. All softness was gone, and the mass felt oval, firm, and less than a hen's egg in size. The whole leg and foot were œdematous, but the knee was not so.—(*British Medical Journal*, June 18, 1859.)

